# A comparative study of nutrient composition, bioactive properties and phytochemical characteristics of *Stauntonia obovatifoliola* flesh and pericarp

**DOI:** 10.3389/fnut.2022.1013971

**Published:** 2022-09-09

**Authors:** Rurui Li, Yuerong Ru, Ling Feng, Zhenxing Wang, Xiahong He, Xuechun Zhang

**Affiliations:** ^1^Key Laboratory for Forest Resources Conservation and Utilization in the Southwest Mountains of China, Ministry of Education, Southwest Forestry University, Kunming, China; ^2^College of Life Science, Southwest Forestry University, Kunming, China; ^3^College of Horticulture and Landscape, Southwest Forestry University, Kunming, China

**Keywords:** *Stauntonia obovatifoliola*, fraction, nutrient composition, antioxidant, α-glucosidase, acetylcholinesterase, HPLC

## Abstract

A comparative study was conducted among the flesh (SOF) and pericarp (SOP) of *Stauntonia obovatifoliola*, a wild edible fruit in China. The nutrient composition of both these tissues was firstly quantified, and liquid-liquid extraction was then used to separate their methanolic extracts to get petroleum ether, chloroform, ethyl acetate, n-butanol, and residual aqueous fractions, which were evaluated for their total phenol content (TPC), total flavonoid content (TFC), antioxidant capacities, and α-glucosidase and acetylcholinesterase inhibition abilities. Finally, high-performance liquid chromatography (HPLC) was used to analyze their phytochemical composition. The results revealed the excellent nutritional properties of both SOF and SOP, especially SOP (total dietary fiber, 15.50 g/100 g; total amino acids, 0.80 g/100 g; vitamin C, 18.00 mg/100 g; Ca, 272.00 mg/kg; K, 402.00 mg/100 g). For both tissues, their ethyl acetate fractions showed the highest TPC (355.12 and 390.99 mg GAE/g DE) and TFC (306.58 and 298.48 mg RE/g DE). Surprisingly, the ethyl acetate fraction of SOP exhibited the strongest DPPH and ABTS radical scavenging capacity with 1046.94 and 1298.64 mg Trolox/g, respectively, which were higher than that of controls Vc and BHT. In contrast, their chloroform fractions exhibited the strongest ferric reducing antioxidant power (1903.05 and 1407.11 mg FeSO_4_/g DE) and oxygen radical absorbance capacity (951.12 and 1510.21 mg Trolox/g DE). In addition, the ethyl acetate fraction of SOF displayed superior α-glucosidase inhibition ability with the IC_50_ value of 0.19 mg/mL, which was comparable to control acarbose. In comparison, the ethyl acetate fraction of SOP had the best acetylcholinesterase inhibition ability with the IC_50_ value of 0.47 mg/mL. The HPLC analysis results demonstrated that the ethyl acetate fraction of SOP showed significantly higher phenolic content, particularly for phenolic acids (p-hydroxybenzoic acid, 8.00 ± 0.65 mg/g) and flavonoids (epicatechin, 28.63 ± 1.26 mg/g), as compared to other samples. The above results suggest that *Stauntonia obovatifoliola*, especially its pericarp, had excellent nutrient compositions, bioactive properties and phytochemical characteristics, and had the potential to be developed as natural functional food.

## Introduction

Reactive oxygen species (ROS) are the highly reactive products of cellular oxygen metabolisms in humans (1), and excessive ROS accumulation may stimulate the cells to produce large amounts of free radicals, which further leads to oxidative stress (OS) related diseases, such as cardiovascular, cancer, diabetes, inflammation, obesity, and Alzheimer's (2). Many studies have indicated that antioxidants could inhibit or prevent OS-induced damage through scavenging free radicals (3, 4). As reported, a range of plants, including fresh fruits, vegetables, nuts, and grains, have shown favorable anti-oxidative stress effects, which are mainly attributed to the abundant active components such as phenols, flavonoids, and alkaloids (5). Compared with synthetic antioxidants, antioxidant ingredients obtained from these plants have the advantage of being natural, safe, and effective, and perform numerous functions for human health. Thus, they have been widely applied in food, medicine, cosmetics, agriculture, and other fields (6, 7).

*Stauntonia obovatifoliola* (SO), which belongs to the genus Akebia Decne (Family Lardizabalaceae), is a perennial woody vine mainly distributed in China (8). SO fruit is composed of the pericarp (SOP) and flesh (SOF), and SOF is the most frequently edible part with yellow color and sweet taste, which accounts for about 60% of the whole fruit (9). It has been documented that SO fruit is a rich source of proteins, pectin, vitamins, phenolics, flavonoids, triterpenoids, and minerals, which confers its excellent medicinal, edible, and ornamental value (10, 11). Especially in the field of folk medicine, SO has shown antipyretic, diuretic, dredge meridians, analgesic, and lactogenic effects, and could be used in the treatment of many diseases, such as rheumatism, beriberi, axillary carbuncle, cystitis, traumatic injury, trigeminal neuralgia, and sciatica (12, 13). Although there are various positive effects on health, SO is still underutilized, only a tiny amount has been consumed as a fruit. Research on the chemical composition and biological activity of SO is also sparse, and its pericarp is normally discarded, which restricts its further application and development.

Generally, the fruit consists of flesh and pericarp. Despite the main edible part of the fruit being flesh, the pericarp also has excellent nutrient composition and bioactivity, which is not inferior to the flesh. As reported by Inoue et al. (14), the pericarp of kiwifruit contains more polyphenols than the flesh. Besides, Zhang et al. (15) also pointed out that the peel of red-fleshed apple had more potent antioxidant activity and a higher total phenolic content than the flesh and whole apple. However, no literature is available on the comparative study between SOP and SOF.

Hence, this study first compared the nutritional composition between SOF and SOP. Then various SO extracts (SOEs) with different polar fractions were obtained by using ultrasound-enzyme-assisted extraction and sequential extraction, and their bioactive properties were investigated, including the total phenolic content, total flavonoid content, antioxidant properties, α-glucosidase and acetylcholinesterase inhibition abilities. Finally, the phytochemical characteristics of SOEs were quantified by using high-performance liquid chromatography (HPLC).

To our knowledge, this is the first study to compare the nutrient composition, bioactive properties, and phytochemical characteristics between SOP and SOF, in special for studies comparing their different polar solvents extracts. This work may provide a scientific basis for finding naturally occurring antioxidants and the further utilization of SO resources.

## Materials and methods

### Standards and reagents

2,2′-azino-bis-(3-ethylbenzothiazoline-6-sulfonic acid) (ABTS), 1,1-diphenyl-2-picrylhydrazylradical (DPPH), 2,4,6-Tri(2-pyridyl)-s-triazine (TPTZ), 2,2′-Azobis(2-amidinopropane) dihydrochloride (AAPH), fluorescein sodium, acetylthiocholine iodide (ATCI), galantamine (GLTM), 5,5′-dithiobis-2-nitrobenzoic acid (DNTB), vitamin C (Vc), 2,6-di-tert-butyl-4-methylphenol (BHT) were obtained from Aladdin Biotechnology (analytical grade, China). While petroleum ether, chloroform, ethyl acetate, and n-Butanol, were purchased from the national medicine group chemical reagent Co., Ltd. (China). Acarbose, 4-methylumbelliferyl-β-D-glucuronide (4-MUG), electric eel acetylcholinesterase (AChE, C3389), and α-glucosidase (G5003) from Saccharomyces cerevisiae (EC 3.2.1.20) were purchased from Sigma-Aldrich (USA). All HPLC standards were purchased from Yuanye Bio-Technology (Shanghai, China). Chromatographic grade reagents, such as acetonitrile, methanol, acetonitrile, and formic acid were purchased from Merck (Darmstadt, Germany).

### Preparation of SO extracts

The *Stauntonia obovatifoliola* (SO) fruits were collected from Ganzhou City of Jiangxi Province (114°94′02′′ N, 25°85′07′′ E), China in November 2020, and were identified by Dr. Ling Feng from the College of Forestry, Southwest Forestry University, China. The mature undamaged SO were selected, and the pericarps (SOP) were carefully separated from the flesh (SOF). A portion of each sample was stored at −80°C directly for subsequent nutritional composition analysis and the rest portion of this sample was freeze-dried and ground into fine powder. The powders were filtered with a 40-mesh sieve, and then immediately stored at −80°C. Before extracting, 100 g fine powders of SOP and SOF were weighed, respectively, and mixed with 70% ethyl alcohol solution (w/v) to obtain a material-liquid ratio of 1:20. Then the pH values of the mixtures were adjusted to 5 ± 0.5, followed by the addition of 0.67% cellulose enzyme (3 U/mg) and 0.05% pectinase (40 U/mg). Ultrasonic extraction was performed for 60 min at 50°C (60 kHz, 500 W), subsequently, the supernatants were collected by centrifugation twice at 2,200 × g for 15 min (TGL-20M, Xiangyi Centrifuge Instrument Co., Ltd., Changsha, China). Afterward, the supernatants were rotating evaporated and made up to 5 L with 70% ethyl alcohol solution (crude extract, CE), while the precipitate was vacuum-dried for later use (residue, RD). The crude extracts were successively extracted with solvents of different polarities, finally obtained petroleum ether (PE), chloroform (CF), ethyl acetate (EtOAc), n-Butanol (n-BuOH) fractions, and aqueous phase residue (AQ), then the extractions were concentrated in vacuo. All SO extracts (SOEs) were stored at −20°C before use.

### Nutrient and non-nutrient composition analyses

Moisture, ash, proteins, fat, carbohydrates, total dietary fiber, amino acids, vitamins, and minerals in the SOF and SOP were determined using accepted analysis methods of the GB5009–2016 of China National Food Safety Standard.

#### The basic nutrients analyses

Moisture was assessed by the oven-drying (16), and ash content was analyzed by incineration technique (17). Fat and dietary fiber contents were determined using the soxhlet extraction method (18) and enzymatic–gravimetric (19), respectively. The %carbohydrate content was calculated by difference method: percentage (%) carbohydrate = 100 – (%Moisture + %ash+ %protein + %crude fiber).

#### Total amino acid and protein contents analyses

The amino acids were determined by national food safety standards (GB 5009.124–2016) (20). Briefly, the SOF and SOP sample was accurately weighed and hydrolysed with hydrochloric acid (6 M) at 110°C for 22 h, after dried under reduced pressure, dissolved in sodium citrate buffer (0.2 M, pH 2.2). Finally, filtered through 0.22 μm membrane and injected into an amino acid analyzer (Hitachi L-8900, Hitachi Ltd., Tokyo, Japan) fitted with sulfonic cation exchange resin. The separated amino acids generated color reaction with ninhydrin solution, absorbance was measured at 440 and 570 nm. The concentration of amino acids was calculated on the basis of standard peak area.

The content of total protein estimated by the Kjeldhal method (GB 5009.5–2016) (21). Briefly, the sample was catalytic heated until fully digested to release ammonia, then connected with sulfuric acid resulting in ammonium sulfate. Ammonia was dissociated by alkalization distillation, boric acid was absorbed and titrated with hydrochloric acid standard solution. Total protein content is calculated based on acid consumption as follows:


(1) $$\begin{array}{r} X=\frac{\left(V_{1}-V_{2}\right)\times c\times 0.014}{m\times V_{3}/100}\times F\times 100 \end{array}$$

X: the total protein amount in sample, g/100 g; *V*_1_: hydrochloric acid consumption volume of samples, mL; *V*_2_: hydrochloric acid consumption volume of blank control, mL; *V*_3_: absorption volume of the digestive liquid, mL; *c*: concentration of hydrochloric acid; *m*: mass of the sample, g; *F*: conversion coefficient of nitrogen to protein.

#### Vitamins and minerals analyses

Vitamin B_1_, B_2_, and vitamin C were determined using the high performance of liquid chromatography (HPLC-2030C plus 3D, Shimadzu, Kyoto, Japan), the quantification was performed by external calibration (GB 5009.84/85/86–2016) (20). The mineral elements of calcium (Ca), magnesium (Mg), potassium (K), sodium (Na), zinc (Zn), iron (Fe), manganese (Mn), and copper (Cu) were determined by atomic absorption spectrometer (Beijing Pu Analysis General Instrument Co., Ltd, Beijing, China) equipped with flame atomization (GB 5009–2016) (19).

### Determination of total phenolic content

The total phenolic content (TPC) was determined according to the Folin-Ciocalteu method (22). Briefly, 50 μL gallic acid solutions (10 to 100 μg/mL) were loaded onto a 96 well microtiter plate, followed by the addition of 125 μL Folin-phenol reagent and 100 μL Na_2_CO_3_ (7.5%, w/v) each well. After incubation at room temperature protected from light for 30 min, the absorbance was read at 760 nm using SPECTRAmax Plus384 microplate Reader. The absorbance was assayed as described above, and the TPC was calculated against a gallic acid standard and was expressed in mg gallic acid equivalents per g dry extract (mg GAE/g DE).

### Determination of total flavonoid content

The total flavonoid content (TFC) was determined by the sodium nitrite-aluminum method (23). 40 μL sample solution and 20 μL NaNO_2_ (3%, w/v) were loaded onto a 96 well microtiter plate, after 6 min of incubation in darkness, 20 μL Al (NO_3_)_3_ solution (6%, w/v) was added and reacted for 6 min, followed by the addition of 140 μL NaOH (4%, w/v) and 60 μL methanol, then the absorbance was measured at 510 nm after 15 min incubation. TFC was calculated against a rutin standard and was expressed in mg rutin equivalents per g dry extract (mg RE/g DE).

### Analysis of antioxidant activity

#### DPPH radical scavenging activity

The DPPH radical scavenging activity was measured by DPPH scavenging assay (24). A 100 μL properly diluted sample solution was mixed with 100 μL of DPPH solution (0.15 mmol/L) on a 96-well plate for 30 min at room temperature in the dark, then the absorbance value was read at 517 nm (*As*). Methanol was used to replace the sample as a negative control (*Ac*), the scavenging rate was calculated by the following formula (1), Trolox (0–25 μg/mL) was used as the standard. Vc and BHT were used as positive controls. The DPPH radical scavenging activity was expressed as mg Trolox equivalents per gram of dry extract (mg Trolox /g DE).


(2) $$\begin{array}{r} \text{DPPH}\;\text{radical}\;\text{scavenging}\;\text{rate}\;\left(\text{\%}\right)=\left[\left(A_{c}-A_{s}\right)/A_{c}\right]\times 100\% \end{array}$$

#### ABTS radical scavenging activity

The ABTS radical scavenging activity was estimated following the reported method (25). A 50 μL appropriate concentration of sample solution was mixed with 200 μL of ABTS solution on a 96-well plate for 6 min at room temperature in the dark, the scavenging rate was the same as formula (2), and the results were expressed as mg Trolox equivalents per gram of dry extract (mg Trolox /g DE). Vc and BHT as positive controls.

#### Ferric reducing antioxidant power

The ferric reducing antioxidant power (FRAP) analysis was performed according to method (26). A 30 μL sample solution with suitable concentration and 240 μL FRAP working solution were loaded onto a 96-well plate, then the absorbance was measured at 593 nm after incubation in darkness for 10 min at 37°C. The FRAP value was calculated by a standard curve using FeSO_4_ as a standard, Vc and BHT as the positive controls, the FRAP value was expressed as mg FeSO_4_ per gram of dry extract (mg FeSO_4_/g DE).

#### Oxygen radical absorbance capacity

The oxygen radical absorbance capacity (ORAC) was carried out as suggested following the reported method (27). A 25 μL sample solution was mixed with 150 μL of fluorescein (8 × 10^−5^ mol/L) on a 96-well plate with a 5 min shaking, after incubated for 10 min at 37 °C, the reaction was initiated with the addition of 50 μL AAPH, then the fluorescence was immediately measured at excitation 485 nm and emission 535 nm excitation every 1 min for 2 h. The ORAC value was calculated using the Area Under the Curve (AUC) with that of the Trolox standard curve and expressed as mg Trolox equivalents per gram of dry extract. Vc and BHT were used as positive controls.

### Enzyme inhibitory ability

#### Inhibitory of α-glucosidase

The α-glucosidase inhibitory activity was measured according to the previous method (28). A 50 μL suitably diluted sample solution was added to 20 μL of α-glucosidase (0.175 U/mL, pH 6.8) mixed well on a black 96-well microplate, followed by addition of 50 μL 4-MUG (0.84 μM, pH 6.8) and incubation 20 min at 37°C. Afterward, a 100 μL glycine (100 mM, pH 10.6) was added to terminate the reaction with a 30 s shaking. The fluorescence was measured at excitation 355 nm and emission 460 nm (*As*), acarbose as the positive control, methanol in place of the sample was used as a reagent blank (*Ab*), and potassium phosphate buffer in place of acetylcholine was used as a negative control (*Ac*). The inhibitory rate of α-glucosidase was calculated by the following formula (3), and the inhibitory capacity expressed as IC_50_ value (mg/mL), which was obtained by plotting scavenging percentage against extract concentration.


(3) $$\begin{array}{r} \text{Inhibition rate }\left(\text{\%}\right)=\frac{A_{b}-\left(A_{s}-A_{c}\right)}{A_{c}}\times 100\% \end{array}$$

#### Inhibition of the acetylcholinesterase

The acetylcholinesterase enzyme (AChE) inhibitory activity was evaluated using Ellman's method (29). A 50 μL appropriate diluted sample solution was added to 90 μL Ellman's reagent (containing 15 μL of 15 mM ATCI and 75 μL of 3 mM DTNB) on a 96-well plate, after incubated for 10 min at 37°C, 20 μL of acetylcholine (0.1 U/mL, in pH 8.0 PBS containing 0.1% bovine serum albumin) was added to initiate the reaction, then absorbance was read at 405 nm after incubation for 5 min in darkness, and galanthamine as positive control. The inhibition rate was calculated in accordance with Equation (3).

### HPLC analysis

The High-performance liquid chromatography with diode array detection (HPLC-DAD) analysis was performed by an Agilent LC 1260 system (Agilent Technologies, CA, USA) (30). All samples redissolved in 1,000 μL of methanol, then were filtered by a 0.22-μm membrane and the injection volume was 20 μL. The gradient elution of the mobile phase contained (A) acetonitrile and (B) water with 0.1% formic acid, and the gradient elution was programmed as follows: 0 min, 5% A; 5 min, 5% A; 7 min, 10% A; 52 min, 30% A; 65 min, 100%A, following by washing with 100% A for 15 min and re-equilibration of the column with 5% A for 10 min. The chromatogram scan range was set to 200–400 nm. The calibration curve was obtained by plotting the peak areas of the standards against their concentrations, five-point calibration curves were prepared using protocatechuic acid (y = 9.4793x - 43.031, *R*^2^ = 0.996), p-hydroxybenzoic acid (y = 19.622x + 14.703, *R*^2^ = 0.996), vanillic acid (y = 42.899x - 808.49, *R*^2^ = 0.995), syringic acid (y = 41.199x - 316.19, *R*^2^ = 0.999), epicatechin (y = 7.0346x + 58.821, *R*^2^ = 0.997), dihydromyricetin (y = 16.512x + 6.3045, *R*^2^ = 0.999), syringaldehyde (y = 41.199x - 316.19, *R*^2^ = 0.999), ferulic acid (y = 9.1787x +1.345, *R*^2^ = 0.992), epigallocatechin gallate (y = 22.222x + 208.9, *R*^2^ = 0.999), ellagic acid (y = 6.4055x - 154.78, *R*^2^ = 0.992), salicylic acid (y = 5.2213x - 61.888, *R*^2^ = 0.997), cinnamic acid (y = 80.95x - 127.56, *R*^2^ = 0.995), hesperetin (y = 34.508x - 1.145, *R*^2^ = 0.999), scutellarin (y = 34.83x - 85.123, *R*^2^ = 0.999), all standards were monitored at 280 nm. Further, the identification and quantification of compounds were made by comparing the retention time and peak area of corresponding standard. The results were expressed as mg equivalents of the standard per g of sample.

## Data analysis and statistics

All tests were repeated 3 times in parallel, and the results were represented as mean ± standard deviation (mean ± SD). Statistical analysis was performed using variance tests analysis of variance (ANOVA) and correlation analysis (*p* < 0.05) with Statistical Package for the Social Sciences (SPSS) 22.0.

## Results

### Nutrient and non-nutrient composition

From Table 1, the nutrition and non-nutrient components of SOF (100 g) were as follows: moisture 82.9 g, ash 0.44 g, protein 0.81 g, fat 0.505 g, carbohydrate 12.91 g, and total dietary fiber 3.4 g. While the SOP (100 g) content was: moisture 79.1 g, ash 0.44 g, protein 0.81 g, fat 0.51 g, and carbohydrate 12.91 g. It could be found that the fat content was reported as the lowest (0.49 g) in SOP, in contrast, which consists of a higher amount of total dietary fiber (15.5 g). Amino acids are the basic units of protein and growth factor and vital nutritional elements in the human body. In terms of amino acid composition (Table 1), the content of total and essential amino acid compositions of SOP was higher than that of the SOF. Moreover, vitamin C (18 mg/100 g) was particularly abundant in SOP, which was an important and frequent antioxidant that could promote collagen regeneration and iron absorption, and prevent scurvy (31).

**Table 1:** The nutrient and non-nutrient composition of SOF and SOP.

	**Concentration**	
**Parameter**	**SOF**	**SOP**
**Nutritional composition**		
Moisture (g/100 g)	82.90 ± 4.14	79.10 ± 3.95
Ash (g/100 g)	0.44 ± 0.02^a^	1.30 ± 0.06^b^
Protein (g/100 g)	0.81 ± 0.04^a^	0.68 ± 0.03^b^
Fat (g/100 g)	0.51 ± 0.02^a^	0.49 ± 0.02^b^
Carbohydrate (g/100 g)	12.91 ± 0.64^a^	3.42 ± 0.17^b^
Total dietary fiber (g/100 g)	3.40 ± 0.17^b^	15.50 ± 0.77^a^
**Amino acids**		
Aspartic acid (g/100 g)	0.10 ± 0.00	0.10 ± 0.00
Threonine (g/100 g)	0.03 ± 0.00^b^	0.05 ± 0.00^a^
Serine (g/100 g)	0.03 ± 0.00^b^	0.05 ± 0.00^a^
Glutamic acid (g/100 g)	0.06 ± 0.00^b^	0.08 ± 0.00^a^
Glycine (g/100 g)	0.03 ± 0.00^b^	0.04 ± 0.00^a^
Alanine (g/100 g)	0.07 ± 0.00^a^	0.05 ± 0.00^b^
Valine (g/100 g)	0.04 ± 0.00^b^	0.06 ± 0.00^a^
Methionine (g/100 g)	0.01 ± 0.00	0.01 ± 0.00
Isoleucine (g/100 g)	0.03 ± 0.00^b^	0.04 ± 0.00^a^
Leucine (g/100 g)	0.04 ± 0.00^b^	0.07 ± 0.00^a^
Tyrosine (g/100 g)	0.02 ± 0.00^b^	0.03 ±0.00^a^
Phenylalanine (g/100 g)	0.03 ± 0.00^b^	0.05 ± 0.00^a^
Lysine (g/100 g)	0.04 ± 0.00^b^	0.06 ± 0.00^a^
Histidine (g/100 g)	0.01 ± 0.00^b^	0.02 ± 0.00^a^
Arginine (g/100 g)	0.03 ± 0.00^b^	0.04 ± 0.00^a^
Proline (g/100 g)	0.04 ± 0.00^b^	0.05 ± 0.00^a^
∑ EAAs	0.18 ± 0.00^b^	0.28 ± 0.01^a^
∑ NEAAs	0.43 ± 0.02^b^	0.52 ± 0.02^a^
∑AA	0.61 ± 0.03^b^	0.80 ± 0.04^a^
∑ EAAs/Total AA (%)	29.00 ±1.45^b^	35.00 ± 1.75^a^
∑ EAAs/∑ NEAAs	0.41± 0.02^b^	0.53 ± 0.02^a^
**Vitamin**		
Vitamin B_1_ (mg/100 g)	ND	ND
Vitamin B_2_ (mg/100 g)	0.14 ± 0.00^a^	ND
Vitamin C (mg/100 g)	ND	18.00 ± 0.90^a^
**Minerals**		
Ca (mg/kg)	125.00 ± 6.25^b^	272.00 ±13.60^a^
Mg (mg/kg)	264.00 ± 13.20^a^	199.00 ± 9.95^b^
K (mg/100 g)	394.00 ± 19.70^b^	402.00 ± 20.10^a^
Na (mg/100 g)	ND	ND
Zn (mg/kg)	ND	ND
Fe (mg/kg)	3.51 ± 0.17^a^	2.25 ± 0.11^b^
Mn (mg/kg)	26.30 ± 1.31^a^	17.70 ± 0.88^b^
Cu (mg/kg)	ND	ND

∑ EAAs, sum of essential amino acids; NEAAs, Non-essential amino acids; ∑ AA, Total Amino acids; ND, not detected. SOF, *Stauntonia obovatifoliola* flesh; SOP, *Stauntonia obovatifoliola* pericarp. The superscript letters indicate the statistical difference in row is significant (*p* < 0.05), without letters indicate not significantly different (*p* > 0.05).

In the present study, both SOP and SOF had abundant essential macro-elements (Ca, Mg, K, Na) and trace elements (Zn, Fe, Mn, Cu). Among them, SOP had a significantly higher concentration of Ca (272 mg/kg, *p* < 0.05), while a higher amount of Mg was observed for SOF (264 mg/kg, *p* < 0.05). For K, there were no significant differences between the two. SOF showed higher levels of Fe and Mn, two vital trace elements for normal function and development of the human body (32), but Zn and Cu were not detected in either SOF or SOP.

This study was similar to what had been reported by Wang et al. (33) but more comprehensive. Overall, both SOF and SOP demonstrated considerable nutritional values, such as well-balanced amino acid composition and minerals, when compared to other common fruits peach apple, pear, quince and apple (31, 34). Of which, SOF had higher energy, and SOP was a good source of dietary fibers. Surprisingly, SOP showed excellent content of Vitamin C, and its content was much higher than that of *Synsepalum dulcificum* berry (20). This revealed that SOP might be a potential source of natural antioxidants and warranted a deeper examination.

### Total phenolic content and total flavonoid content

As described in the Methods Section Preparation of SO extracts, there were 7 SO extracts (SOEs) for SOF and SOP, respectively, which were in turn crude extract (CE), extract residue (RD), petroleum ether fraction (PE), chloroform fraction (CF), ethyl acetate fraction (EtOAc), n-Butanol fraction (n-BuOH), and aqueous phase residue (AQ). According to Table 2, all SOEs of SOP showed higher total phenolic content (TPC) than that of SOF, while the EtOAc fractions were significantly highest in both groups with values of 390.99 ± 3.72 and 355.12 ± 10.39 mg GAE/g DE (*p* < 0.05). That was proposed to be related to the abundant tannin in SOP (12, 35).

**Table 2:** Total phenolic content and total flavonoid content of SOEs.

**Extracts**	**TPC (mg GAE/g DE)**		**TFC (mg RE/g DE)**	
	**SOF**	**SOP**	**SOF**	**SOP**
CE	219.24 ± 15.13^b^	247.64 ± 7.75^b^	169.48 ± 5.51^c^	189.61 ± 5.50^b^
PE	15.26 ± 1.54^e^	31.38 ± 3.39^e^	11.34 ± 2.15^e^	24.06 ± 1.48^e^
CF	187.93 ± 8.12^c^	199.37 ± 4.29^c^	135.60 ± 0.93^b^	148.41 ± 6.15^c^
EtOAc	355.12 ± 10.39^a^	390.99 ± 3.72^a^	306.58 ± 8.77^a^	298.48 ± 3.59^a^
n-BuOH	118.98 ± 4.10^c^	138.70 ± 10.24^d^	94.84 ± 2.16^d^	94.67 ± 12.8^d^
AQ	24.18 ± 1.57^d^	28.43 ± 0.15^e^	20.76 ± 1.18^e^	2.75 ± 1.49^e^
RD	18.94 ± 0.69^e^	26.66 ± 2.84^e^	15.54 ± 1.29^e^	21.65 ±3.60^e^

SOEs, *Stauntonia obovatifoliola* extracts; SOF, *Stauntonia obovatifoliola* flesh; SOP, *Stauntonia obovatifoliola* pericarp; GAE, gallic acid equivalent; RE, rutin equivalent; TPC, total phenolic content; TFC, total flavonoid content; CE, crude extract; PE, Petroleum ether fraction; CF, chloroform fraction; EtOAc, ethyl acetate fraction; n-BuOH, n-Butanol fraction; AQ, aqueous phase residue; RD, residue. The superscript letters indicate the statistical difference in columns is significant, *p* < 0.05.

Similar to TPC, the highest total flavonoid contents (TFC) were also observed in the EtOAc fractions, whether SOF or SOP, and these values were 306.58 ± 8.77 and 298.48 ± 3.59 mg RE/g DE. Interestingly, with the exception of CE and CF (SOP > SOF, *p* < 0.05), no significant variation was observed for other SOEs. These results were in accordance with the research that the SOP contained significantly more phenolic and flavonoid compounds than SOF overall (10).

### Antioxidant properties

**Figure 1:**
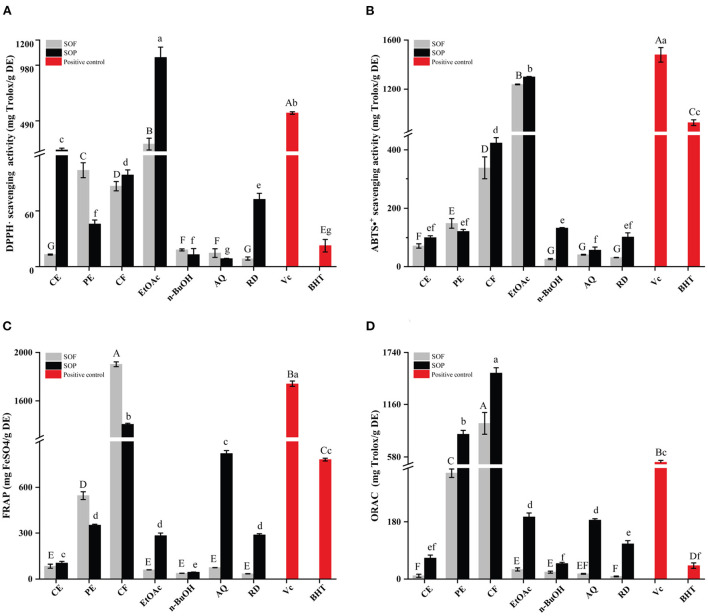
The antioxidant activities of the SOEs and its fractions. **(A)** DPPH· scavenging activity (DPPH), **(B)** ABTS·^+^ scavenging ability (ABTS), **(C)** Ferric reducing antioxidant power (FRAP), **(D)** Oxygen radical absorbance capacity (ORAC). SOEs, *Stauntonia obovatifoliola* extracts; SOF, *Stauntonia obovatifoliola* flesh; SOP, *Stauntonia obovatifoliola* pericarp. CE, crude extract; PE, Petroleum ether fraction; CF, chloroform fraction; EtOAc, ethyl acetate fraction; n-BuOH, n-Butanol fraction; AQ, aqueous phase residue; RD, residue. The superscript letters indicate the statistical difference in rows in significant, *p* < 0.05.

Due to the different action mechanisms, the antioxidant capabilities of SOEs were evaluated by four assays (radical scavenging capacity for DPPH and ABTS, FRAP, and ORAC) (36). Furthermore, the CF, PE, RD, and CE fractions of SOF and SOP also showed strong DPPH inhibitory activities compared with BHT (Figure 1A).

Similar to DPPH radical scavenging activities, the EtOAc fractions of SOF and SOP also exhibited the highest ABTS radical scavenging activities (Figure 1B), with the values of 1237.41 ± 3.30 and 1298.64 ± 3.56 mg Trolox/g, which were lower than Vc (1479.39 ± 52.27 mg Trolox/g DE, *p* < 0.05) and stronger than BHT (922.84 ± 23.12 mg Trolox/g DE, *p* < 0.05). Overall, there is little difference between SOF and SOP, but varied markedly between different extraction solvents.

The ferric reducing antioxidant power (FRAP) of all samples were illustrated in Figure 1C. Interestingly, the highest FRAP values were reported for the CF fractions of SOF and SOP (1903.05 ± 20.07 and 1407.11 ± 9.33 mg FeSO_4_/g DE), than that of the EtOAc fraction of SOF or SOP. The CF fraction of SOF was stronger than V_C_ (1741.74 ± 23.19 mg FeSO_4_/g DE, *p* < 0.05), even reached 2-fold of BHT (781.91 ± 9.47 mg FeSO_4_/g DE). Among all of the SOEs, the n-BuOH fraction of SOF or SOP exhibited the lowest activity (37.38 ± 0.54 and 44.21 ± 0.87 mg FeSO_4_/g DE).

The oxygen radical absorbance capacity (ORAC) assay is a widely accepted method of measuring the antioxidant capacity of different biological samples, and is considered to be associated with health benefits (27). As shown in Figure 1D, the CF extracts of SOP and SOF possessed the highest ORAC with the values of 1510.21 ± 60.39 and 951.12 ± 120.58 mg Trolox/g DE, followed by the PE fraction of SOP (831.65 ± 41.27 mg FeSO_4_/g DE), and the above three SOEs were significantly higher than Vc (519.69 ± 21.89 mg Trolox/g DE). Overall, SOP displayed stronger ORAC than SOF for each fraction, and the CE, EtOAc, AQ, and RD fractions of SOP all exhibited higher ORAC values than BHT (41.62 ± 8.31 mg Trolox/g DE).

**Figure 2:**
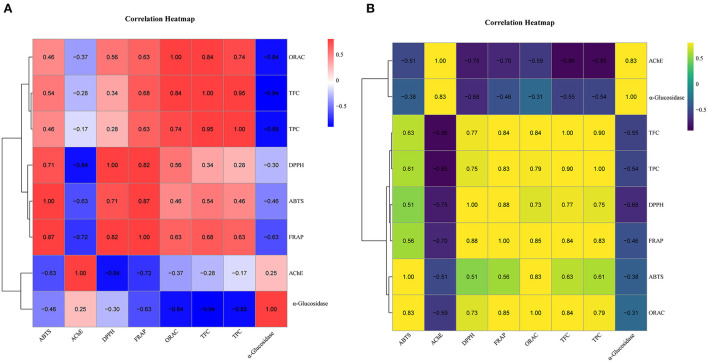
The correlational analyses of experiment. **(A)** Heatmap of SOF correlation. **(B)** Heatmap of SOP correlation. The color represents the magnitude of correlation (−1 to 1). TPC, total phenolic content; TFC, total flavonoid content; DPPH, DPPH·scavenging activity; ABTS, ABTS·^+^ scavenging ability; FRAP, the ferric reducing antioxidant power; ORAC, oxygen radical absorbance capacity; α-glucosidase, the IC_50_ value for α-glucosidase inhibitory; AChE, the IC_50_ value for acetylcholinesterase inhibitory.

Further, we performed the correlation analysis among the various indicators of SOF and SOP separately (Figure 2). Overall, all four antioxidant activities were positively correlated with TPC and TFC. This also supported the view that, in many cases, the antioxidant activities of plant extracts were correlated with their total phenolics and flavonoid contents (37). Compared to SOF, these activities of SOP processed higher association with TPC (0.61 < *r* < 0.83, *p* < 0.05) and TFC (0.63 < *r* < 0.84, *p* < 0.05), while the correlation coefficients between these antioxidant activities of SOF and TPC were 0.28 to 0.74, and 0.34 to 0.84 with TFC. Among these four antioxidant activities, FRAP and ORAC displayed more significantly correlated with other indicators, which illustrated that these two assays could give a broader antioxidant characterization to SO. Together with the results shown in earlier section Total phenolic content and total flavonoid content, our data indicated that pericarp tissues contained a larger number of phenolics and flavonoids than flesh, which might be the main contributors to these antioxidant activities.

**Figure 3:**
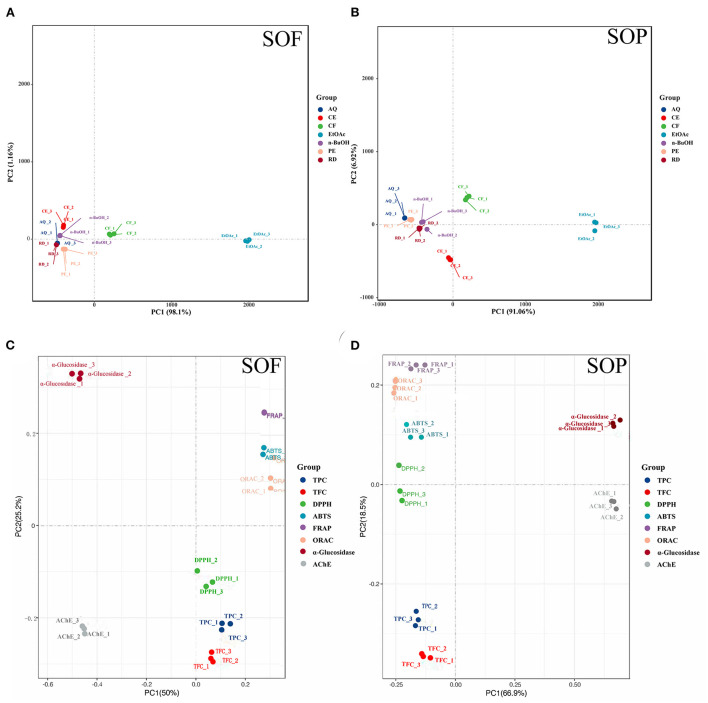
Principal component analysis (PCA) of different samples and indicators. **(A,B)** PCA of seven SOEs and fractions; **(C,D)** principal co-ordinates analysis (PCoA) of eight indicators of active ingredient content and biological activity; SOEs, *Stauntonia obovatifoliola* extracts; SOF, *Stauntonia obovatifoliola* flesh; SOP, *Stauntonia obovatifoliola* pericarp. CE, crude extract; PE, Petroleum ether fraction; CF, chloroform fraction; EtOAc, ethyl acetate fraction; n-BuOH, n-Butanol fraction; AQ, aqueous phase residue; RD, residue; TPC, total phenolic content; TFC, total flavonoid content; DPPH, DPPH· scavenging activity; ABTS, ABTS·^+^ scavenging ability; FRAP, the ferric reducing antioxidant power; ORAC, oxygen radical absorbance capacity; α-glucosidase, the IC_50_ value for α-glucosidase inhibitory; AChE, the IC_50_ value for acetylcholinesterase inhibitory.

Further principal component analysis (PCA) revealed the relationships between different SOEs, and the distances between the samples in the principal component coordinates reflected the difference and correlation between them. From Figures 3A,B, the EtOAc and CF fractions were far from other fractions, and all fractions were also easily distinguishable, which verified the experimental results of antioxidant assays that EtOAc and CF fractions showed more excellent antioxidant activity compared to other SOEs. Principal coordinates analysis (PCoA) showed the clustering results between different indicators (Figures 3C,D). We observed the close distances between TPC, TFC, and four antioxidant activities at PC1, but they were clearly separated across PC2, and SOP was better separated. This was also consistent with the above results of correlation analysis.

### Inhibitory of α-glucosidase

**Figure 4:**
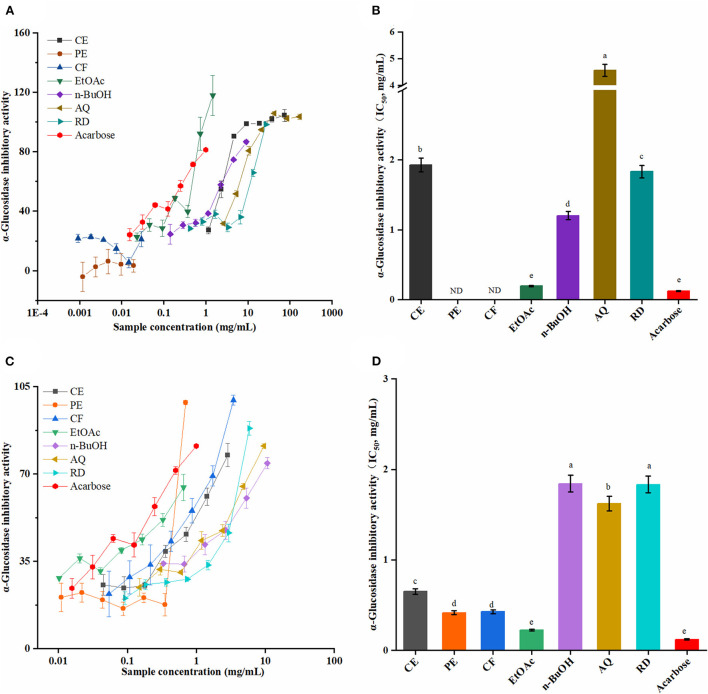
The α-glucosidase inhibitory activities of the SOEs. **(A,C)** The inhibition ratio of α-glucosidase incubated with different concentrations of SOF and SOP; **(B,D)** The IC_50_ values of SOF and SOP for α-glucosidase inhibition. SOEs, *Stauntonia obovatifoliola* extracts; SOF, *Stauntonia obovatifoliola* flesh; SOP, *Stauntonia obovatifoliola* pericarp. CE, crude extract; PE, Petroleum ether fraction; CF, chloroform fraction; EtOAc, ethyl acetate fraction; n-BuOH, n-Butanol fraction; AQ, aqueous phase residue; RD, residue; ND, not detected; IC_50_, half maximal inhibitory concentration. The superscript letters indicate the statistical difference in rows in significant, *p* < 0.05.

It is well known that α-glucosidase inhibitors, such as acarbose, could restrict the oligosaccharides and disaccharides in the food to be hydrolyzed into monosaccharides, and, in turn, improve fasting glucose and postprandial blood glucose (38). As shown in Figure 4, the SOEs inhibited α-glucosidase in a dose-dependent manner. Among these, the EtOAc fraction of SOF and SOP showed excellent inhibitory activities with the IC_50_ values of 0.19 ± 0.01 and 0.22 ± 0.09 mg/mL, which were close to the antidiabetic drug acarbose (0.12 ± 0.11 mg/mL). Furthermore, the PE and CF fractions of SOP exhibited strong α-glucosidase inhibitory activity, and these IC_50_ values were 0.41 ± 0.02 and 0.42 ± 0.02 mg/mL. These results proved that SOF and SOP had certain effects, especially their EtOAc fractions, which could be developed into α-glucosidase inhibitor drugs.

From Figure 2, correlation analyses revealed that the phenolics and flavonoids compounds in SOF and SOP were the major contributing factors for the α-glucosidase inhibitory activity, of which the α-glucosidase inhibitory activities of the SOEs of SOF showed the highest coefficient correlations with TPC and TFC (*r* = −0.85 and−0.94, *p* < 0.01). Interestingly, although the α-glucosidase inhibitory activities in SOF and SOP both exhibited significant correlations with the antioxidant activities (*p* < 0.01), an obvious difference was found between SOF and SOP. These results indicated that the active ingredients which play critical roles in the inhibition of α-glucosidase and antioxidation were not the same in SOF and SOP, and this also explained the differences in these indicators between them.

### Acetylcholinesterase inhibitory ability

Alzheimer's disease (AD) is the most common progressive neurodegenerative brain disorder featuring memory loss and cognitive impairments in older people with no effective treatment available currently (39). There is a widespread notion that inhibition of AChE could prevent the metabolic processes of acetylcholine (ACh), a neurotransmitter that supports cognitive function in the cerebral cortex and hippocampus, which in turn enhances the ACh levels and delays cognitive decline. Therefore, AChE inhibitors have been the main strategy followed for the treatment of AD (40).

**Figure 5:**
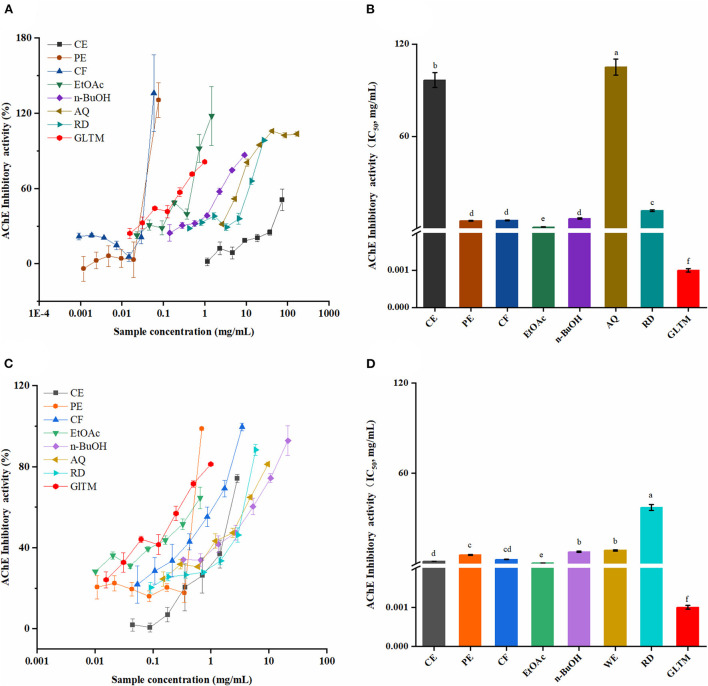
The AChE inhibitory activities of the SOEs. **(A,C)** The inhibition ratio of AChE incubated with different concentrations of SOF and SOP; **(B,D)** The IC_50_ values of SOF and SOP for AChE inhibition; SOEs, *Stauntonia obovatifoliola* extracts; SOF, *Stauntonia obovatifoliola* flesh; SOP, *Stauntonia obovatifoliola* pericarp. CE, crude extract; PE, Petroleum ether fraction; CF, chloroform fraction; EtOAc, ethyl acetate fraction; n-BuOH, n-Butanol fraction; AQ, aqueous phase residue; RD, residue; GLTM, galantamine; IC_50_, half maximal inhibitory concentration. The superscript letters indicate the statistical difference in rows in significant, *p* < 0.05.

The AChE inhibitory activities of SOF and SOP were shown in Figure 5. Similar to the α-glucosidase inhibitory activity, the AChE activity was significantly inhibited by all SOEs in a dose-dependent manner, and SOP processed stronger inhibitory ability compared to SOF.

Among these, the EtOAc fractions of SOP and SOF had the best AChE inhibitory abilities with the IC_50_ values of 0.47 ± 0.02 and 1.04 ± 0.05 mg/mL. Although the inhibitory abilities were lower than that of the positive control galantamine (1 ± 0.01 μg/mL), they were stronger than that of *Chamaerops humilis* L. (40) and *Corchorus depressus* (37). Furthermore, the CE and CF fractions of SOP also showed certain inhibitory ability, and their IC_50_ values were 1.56 ± 0.07 and 2.76 ± 0.13 mg/mL, respectively.

Next, we conducted a correlation analysis. For SOP, its AChE inhibitory was strongly correlated to TFC and TPC (*r* = −0.86 and −0.85, *p* < 0.01), DPPH radical scavenging activity (*r* = −0.75, *p* < 0.01), and α-glucosidase inhibitory (*r* = 0.83, *p* < 0.01), while SOF was relatively low correlated to these indicators, and was clearly distinct from SOP. The correlation analyses above were consistent with the above conclusion of α-glucosidase inhibitory, that the active ingredients in SOP and SOF are not consistent, resulting in differences in their activities such as α-glucosidase and AChE inhibitory abilities (41).

### HPLC analyses

**Figure 6:**
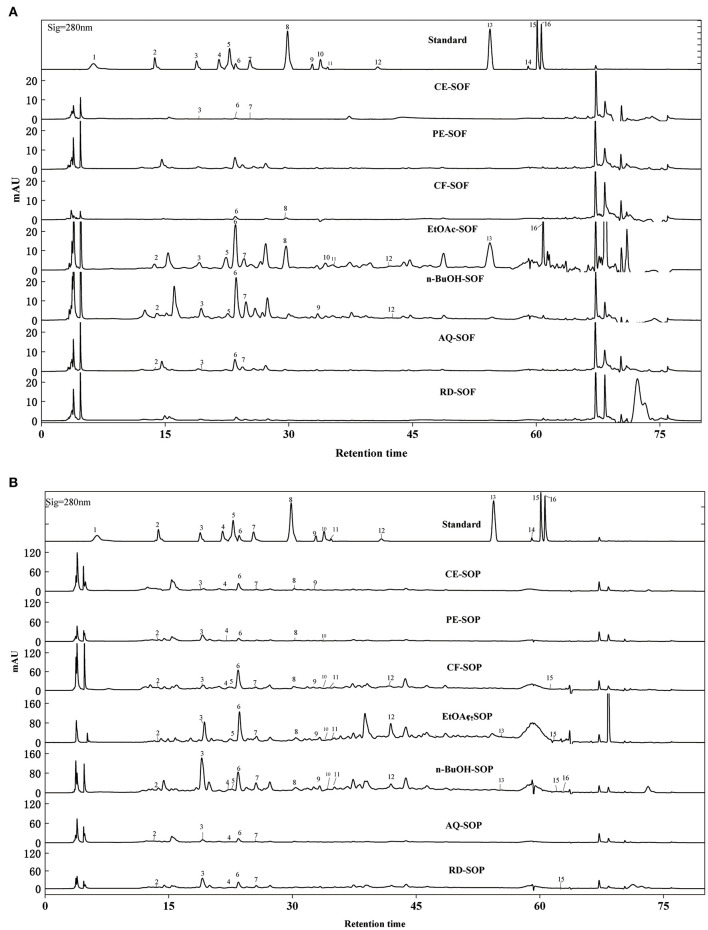
HPLC-DAD chromatogram of SOEs. **(A)** The HPLC chromatogram of SOF extraction phase. **(B)** The HPLC chromatogram of SOP extraction phase. SOEs, *Stauntonia obovatifoliola* extracts; SOF, *Stauntonia obovatifoliola* flesh; SOP, *Stauntonia obovatifoliola* pericarp; mAU, milli-arbitrary units. Detection at 280 nm: (1) Gallic acid, (2) Protocatechuic acid, (3) P-hydroxybenzoic acid, (4) Vanillic acid, (5) Syringic acid, (6) Epicatechin, (7) Dihydromyricetin, (8) Syringaldehyde, (9) Ferulic acid, (10) Epigallocatechin gallate, (11) Ellagic acid, (12) Salicylic acid, (13) Cinnamic acid, (14) Apigenin, (15) Hesperetin, (16) Scutellarin. CE, crude extract; PE, Petroleum ether fraction; CF, chloroform fraction; EtOAc, ethyl acetate fraction; n-BuOH, n-Butanol fraction; AQ, aqueous phase residue; RD, residue.

The qualitative and quantitative analyses were carried out using HPLC-DAD, and the results were presented in Figure 6 and Table 3. Figure 6 showed the similar shapes of the HPLC chromatogram for SOF and SOP, and revealed the differences in their peak intensities. It can be found that the peak intensity for all SOEs was higher for the SOP compared with the SOF group. In addition, the EtOAc fractions of SOF and SOP both had the most chromatographic peaks and the most intense peaks, followed by the n-BuOH and CF fractions.

**Table 3:** The content of individual phenolics from SOF and SOP different fractions.

	**RT (time)**	**Phenolic Compounds (mg/g)**	**Classification**	**Sample**	**Fractions**						
					**CE**	**PE**	**CF**	**EtOAc**	**n-BuOH**	**AQ**	**RD**
1	13.741	Protocatechuic acid	Phenolic acids	Flesh	ND	ND	ND	1.01 ± 0.08^bc^	0.73 ± 0.12^c^	1.62 ± 0.27^b^	ND
				Pericarp	ND	0.92 ± 0.02^bc^	1.24 ± 0.08^bc^	2.97 ± 0.09^a^	3.44 ± 0.19^a^	0.77 ± 0.02^bc^	1.08 ± 0.31b^c^
2	18.812	P-hydroxybenzoic acid	Phenolic acids	Flesh	0.16 ± 0.01^e^	ND	ND	0.47 ± 0.10^de^	0.45 ± 0.01^de^	0.39 ± 0.11^de^	ND
				Pericarp	0.44 ± 0.04^de^	2.37 ± 0.18^d^	1.61 ± 0.16^de^	8.00 ± 0.65^b^	20.7 ± 42.72^a^	0.80 ± 0.05^de^	5.27 ± 1.81^c^
3	21.531	Vanillic acid	Phenolic acids	Flesh	ND	ND	ND	ND	ND	ND	ND
				Pericarp	2.42 ± 0.01^c^	2.19 ± 0.00^b^	2.71 ± 0.03^a^	ND	ND	2.18 ± 0.02^c^	2.19 ± 0.01^b^
4	22.804	Syringic acid	Phenolic acids	Flesh	ND	ND	ND	0.83 ± 0.04^b^	0.47 ± 0.03^c^	ND	ND
				Pericarp	ND	ND	1.02 ± 0.03^b^	0.97 ± 0.08^bc^	1.41 ± 0.43^b^	2.68 ± 0.15^a^	ND
5	24.141	Epicatechin	Phenolic acids	Flesh	0.68 ± 0.02^d^	ND	2.09 ± 0.06^d^	7.09 ± 0.82^c^	5.60 ± 0.22^c^	1.92 ± 0.29^d^	ND
				Pericarp	5.26 ± 0.17^c^	2.16 ± 0.07^d^	16.75 ± 0.18^b^	28.63 ± 1.26^d^	23.90 ± 3.63^a^	0.10 ± 0.07^d^	1.08 ± 0.46^d^
6	25.280	Dihydromyricetin	Flavonoids	Flesh	0.49 ± 0.02^bc^	ND	ND	0.63 ± 0.13^bc^	1.02 ± 0.44^bc^	0.17 ± 0.07^c^	ND
				Pericarp	0.22 ± 0.07^c^	ND	0.77 ± 0.08b^c^	0.79 ± 0.02^bc^	6.00 ± 1.23^a^	ND	1.17 ± 0.25^b^
7	29.842	Syringaldehyde	Flavonoids	Flesh	ND	ND	0.06 ± 0.00^c^	0.38 ± 0.04^a^	ND	ND	ND
				Pericarp	0.04 ± 0.00^c^	ND	0.11 ± 0.00^c^	0.04 ± 0.00^b^	0.59 ± 0.12^b^	ND	ND
8	32.847	Ferulic acid	Phenolic acids	Flesh	ND	ND	ND	ND	0.45 ± 0.03^b^	ND	ND
				Pericarp	0.39 ± 0.04^b^	ND	0.84 ± 0.22^b^	1.35 ± 0.24^b^	5.13 ± 1.94^a^	ND	0.91 ± 0.24^b^
9	33.834	Epigallocatechin gallate	Flavonoids	Flesh	ND	ND	ND	0.24 ± 0.02^b^	ND	ND	ND
				Pericarp	ND	0.14 ± 0.01^c^	0.29 ± 0.07^c^	1.41 ± 0.23^b^	2.43 ± 0.88^a^	ND	0.62 ± 0.15^bc^
10	34.825	Ellagic acid	Phenolic acids	Flesh	ND	ND	ND	3.58 ± 0.05^b^	ND	ND	ND
				Pericarp	ND	ND	3.47 ± 0.06^b^	3.57 ± 0.14^b^	7.99 ± 0.71^a^	ND	ND
11	40.75	Salicylic acid	Phenolic acids	Flesh	ND	ND	ND	2.15 ± 0.06^c^	1.02 ± 0.04^e^	ND	ND
				Pericarp	ND	ND	1.85 ± 0.09^d^	3.23 ± 0.15^a^	2.33 ± 0.02^b^	ND	ND
12	54.392	Cinnamic acid	Phenolic acids	Flesh	ND	ND	ND	0.86 ± 0.07^a^	ND	ND	ND
				Pericarp	ND	ND	ND	0.87 ± 0.17^a^	0.31 ± 0.00^b^	ND	ND
13	60.121	Hesperetin	Flavonoids	Flesh	ND	ND	ND	ND	ND	ND	ND
				Pericarp	ND	ND	0.48 ± 0.11^c^	6.36 ± 1.04^a^	3.02 ± 0.62^b^	ND	0.97 ± 0.09^c^
14	60.622	Scutellarin	Flavonoids	Flesh	ND	ND	ND	1.20 ± 0.08^b^	ND	ND	ND
				Pericarp	ND	ND	ND	ND	2.21 ± 0.14^a^	ND	ND

All values are expressed as “mg/g”, mean ± standard deviation (n = 3). The different letters represent significant difference in the same compound in the *Stauntonia obovatifoliola* flesh and pericarp (*p* < 0.05). RT, retention time; ND, not detected; CE, crude extract; PE, Petroleum ether fraction; CF, chloroform fraction; EtOAc, ethyl acetate fraction; n-BuOH, n-Butanol fraction; AQ, aqueous phase residue; RD, residue.

By comparing retention times and the UV absorbance spectra with the chemical standards, a total of 14 compounds were confirmed and annotated in those chromatograms. Among these, seven phenolic acids were identified in the EtOAc fraction of SOF (Table 3), including epicatechin (7.09 ± 0.82 mg/g), ellagic acid (3.58 ± 0.05 mg/g), salicylic acid (2.15 ± 0.06 mg/g), protocatechuic acid (1.01 ± 0.08 mg/g), cinnamic acid (0.86 ± 0.07 mg/g), syringic acid (0.83 ± 0.04 mg/g), and p-hydroxybenzoic acid (0.47 ± 0.10 mg/g), respectively. While no compounds were detected in the PE and RD fraction of SOF (Figure 6A).

For SOP, nine phenolic acids and five flavonoids were quantified in the EtOAc fraction, and the most abundant phenolic was epicatechin (28.63 ± 1.26 mg/g), which was significantly higher than that in the SOF (*p* < 0.05). Overall, the CE, CF, EtOAc, and n-BuOH fractions of SOP contained more epicatechin, and the PE fraction was most abundant in terms of P-hydroxybenzoic acid, while the AQ and RD fractions were richer in syringic acid (Figure 6B). Interestingly, ferulic acid was only detected in the EtOAc fraction of SOP, but not the EtOAc fraction of SOF, whereas scutellarin appeared only in the EtOAc fraction of SOF but not the EtOAc fraction of SOP. Our result was consistent with the previous literature that pericarp contained more flavonoids as compared to the flesh (42).

**Figure 7:**
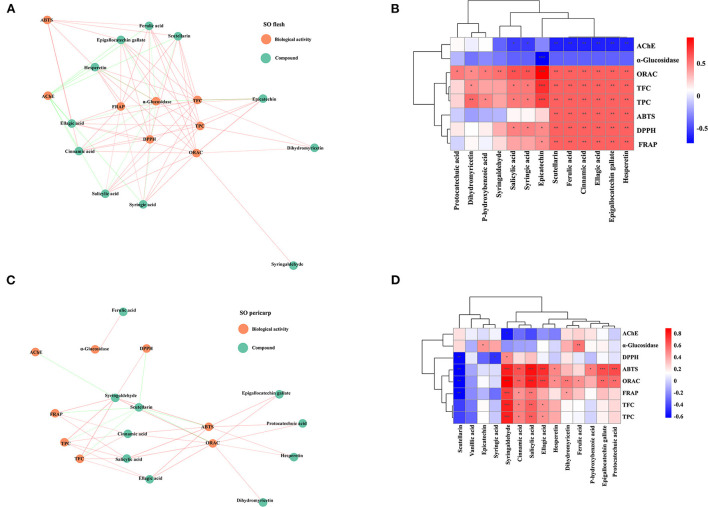
The correlation with compounds from crude extracts and fractions of SO and their biological activities. **(A)** Correlation heat map of SO flesh; **(B)** Correlation network diagram of SO flesh; **(C)** Correlation heat map of SO pericarp; **(D)** Correlation network diagram of SO pericarp. Each line connects the bioactivity or interphase of the compound *p* < 0.05. P-value (ranging from −1 to 1) and corresponding color (blue to red), *p* < 0.05 was considered as significant (*), *p* < 0.01 as highly significant (**), and *p* < 0.001 as very significant (***). SO, *Stauntonia obovatifoliola*; TPC, total phenolic content; TFC, total flavonoid content; DPPH, DPPH· scavenging activity; ABTS, ABTS·^+^ scavenging ability; FRAP, the ferric reducing antioxidant power; ORAC, oxygen radical absorbance capacity; α-glucosidase, the IC_50_ value for α-glucosidase inhibitory; AChE, the IC_50_ value for acetylcholinesterase inhibitory.

Our results indicated that both SOF and SOP are the potential sources of phenolics, in particular the EtOAc fraction of SOP. To understand the relationship between these compounds and biological activities more clearly, a radial network diagram was plotted (Figures 7A,C). The network diagram indicated that these bioactive activities of SO were differentially affected by multiple compounds, and this is further evidenced by the heat maps (Figures 7B,D). For SOF, its TPC, TFC, and antioxidant capacities were significantly and positively correlated with hesperetin, epigallocatechin gallate, ellagic acid, cinnamic acid, and scutellarin. In addition, the IC_50_ values of α-glucosidase and AChE inhibitory activities of SOF were significantly correlated with all of the active substances (*p* < 0.01), which meant that these compounds all contributed significantly to α-glucosidase and AChE inhibitory activities. Of these, it was found that nearly all active indicators in SOP were highly and positively correlated with syringaldehyde, a lignin-derived aromatic compound that was reported in some plant species such as *Manihot esculenta* and *Magnolia officinalis*, and showed antioxidant and anti-inflammatory activities (43, 44).

## Discussion

It is well known that the nutritional value and biological activities differed between the various plant parts, and the biological activities such as antioxidant activity were strongly dependent on the extraction solvent (45). The SO's current research mostly focuses on its germplasm resources and medicinal value, studies on its nutrient and chemical components and bioactivities of different solvent extracts or prepared from various parts of SO were scarcely evaluated.

In this study, we firstly compared the nutritional composition of SOF and SOP, then they were extracted using methanol solution and isolated by liquid-liquid partitioning, respectively. Finally, the bioactive properties and phytochemical characteristics of these fractions were investigated.

Results showed that both SOF and SOP had excellent nutritional properties, including a large amount of dietary fiber, amino acids, vitamins, and minerals, especially SOP. Considering that SOP was often discarded in the harvesting process, it was necessary to be further researched and developed.

The analysis of the chemical compositions and biological activities revealed the substantial variation between these fractions of SOF and SOP. Overall, SOP contained higher TPC and TFC, and it possessed stronger bioactivities, including DPPH and ABTS radical scavenging activities, ORAC, and α-glucosidase inhibitory ability, while SOF performed better in FRAP and AChE inhibitory ability. The reason may be that SOP had more kinds and higher abundances of active compounds than SOF overall, so the majority of the functional activities of SOP were more active (45). But not all functional activities were determined by these compounds, some other compounds which were significantly correlated with FRAP and AChE inhibitory ability, such as hesperidin, epiallocatechin gallate, elliptic acid, cinmic acid, and scutellarin, were found to be higher in SOF, thereby resulting in the difference in these abilities between SOF and SOP.

Of these fractions, the EtOAc fractions of SOF and SOP had the highest TPC and TFC, and possessed the strongest DPPH and ABTS radical scavenging activities, and α-glucosidase and AChE inhibitory abilities. Besides this, the CF fractions of SOF and SOP exhibited the strongest FRAP and ORAC. We attributed this to the following reasons. Firstly, ethyl acetate is proved to be the best extraction solvent to enrich the phenolics and flavonoids compounds, which are the main contributors to these activities (46). In addition, the polarities of EtOAc and CF are closer, while the solvent with similar polarity is easier to get compounds with similar structures, which tend to have similar biological properties. This explains why the CF fraction also possesses good activity (47).

Due to the high concentration of active compounds present in SO, the above fractions even exhibited stronger antioxidant capacity than the positive controls Vc and BHT in some of the indicators, or the inhibitory ability comparable to the commercial drug acarbose. Thus, in contrast to some common plants or fruits such as grape skin (48) and hawthorn fruit (49), SO, especially the EtOAc fraction of SOP, demonstrated the potential strengths of antioxidation, and prevention and treatment of diabetes and AD *in vitro*.

The contents of the major phenolic acid and flavonoid compounds in SO were determined *via* HPLC analysis, and the results indicated that both SOF and SOP are the potential sources of phenolics, in particular the EtOAc fraction of SOP. Overall, SOP contained many more compound kinds and higher compound amounts compared to SOF, this might also explain their bioactivity divergence. Correlation analysis further confirmed the key active compounds for SO, such as hesperetin, epigallocatechin gallate, ellagic acid, cinnamic acid, scutellarin, and syringaldehyde. They were significantly positively correlated with DPPH and ABTS radical scavenging activities, FRAP, and ORAC, which were attributed to the antioxidant activities of phenolic acids and flavonoids. Further, many compounds in SO showed a significant correlation with the α-glucosidase and AChE inhibitory activities, suggesting its hypoglycemic potential and the feasibility of treating AD (21). This could provide further insights into future research on SO.

In conclusion, although there have been some reports on the active substances in SO, such as flavonoids, sterols, phenols, and saponins (9), this is no report to compare the bioactive compounds of its fractions with different polarities. Due to the HPLC detection limit, structural characterization by mass spectrometry is needed for in-depth analysis.

## Conclusions

This study is the first comparative study on the nutrient composition, bioactive properties, and phytochemical characteristics of SOF and SOP. The results showed that both of these possessed excellent nutrient compositions, and SOP had higher nutritional value. Among their different fractions by solvent-solvent extraction, the EtOAc fraction of SOP exhibited excellent bioactive properties and phytochemical characteristics, which demonstrated that *Stauntonia obovatifoliola* had the potential to be developed as natural functional food. However, there are currently insufficient studies on *Stauntonia obovatifoliola*, further comprehensive analysis of its chemical components, *in vivo* bioactivity, and the mechanism of action are needed.

## Data availability statement

The original contributions presented in the study are included in the article/supplementary material, further inquiries can be directed to the corresponding authors.

## Author contributions

RL and ZW: methodology. ZW and YR: validation and writing—review and editing. YR and XZ: formal analysis. RL: data curation and writing—original draft preparation. LF: supervision. XZ and XH: project administration. XH: funding acquisition. All authors have read and agreed to the published version of the manuscript.

## Funding

This work was supported by China Agriculture Research System of MOF and MARA (CARS-21), Major Science and Technology Project of Yunnan and Kunming (202102AE090042, 202204BI090003, 202205AF150018, and 2021JH002), National and Provincial Excellent Scientist Supporting Programme, Yunnan Zhengwenjie Expert Workstation (202205AF150018), Yunnan Special General Projects of Basic Research (202201AT070049), and Yunnan Agricultural Joint Special General Project (202101BD070001-089 and 202101BD070001-109).

## Conflict of interest

The authors declare that the research was conducted in the absence of any commercial or financial relationships that could be construed as a potential conflict of interest.

## Publisher's note

All claims expressed in this article are solely those of the authors and do not necessarily represent those of their affiliated organizations, or those of the publisher, the editors and the reviewers. Any product that may be evaluated in this article, or claim that may be made by its manufacturer, is not guaranteed or endorsed by the publisher.

## References

[1] Aguirre-Joya JA Chacón-Garza LE Valdivia-Najár G Arredondo-Valdés R Castro-López C Ventura-Sobrevilla JM. Nanosystems of plant-based pigments and its relationship with oxidative stress. Food Chem Toxicol. (2020) 143:111433. 10.1016/j.fct.2020.111433 32569796

[2] Gómez GI Velarde V Sáez JC. Role of a rhoa/rock-dependent pathway on renal connexin43 regulation in the angiotensin ii-induced renal damage. Int J Mol Sci. (2019) 20:4408. 10.3390/ijms20184408 31500276 PMC6770162

[3] Kolniak-Ostek J Kłopotowska D Rutkowski KP Skorupińska A Kruczyńska DE. Bioactive compounds and health-promoting properties of pear (pyrus communis l) fruits. Molecules. (2020) 25:4444. 10.3390/molecules25194444 32992651 PMC7582546

[4] Galasso C Piscitelli C Brunet C Sansone C. New *in vitro* model of oxidative stress: Human prostate cells injured with 2,2-diphenyl-1-picrylhydrazyl (dpph) for the screening of antioxidants. Int J Mol Sci. (2020) 21:8707. 10.3390/ijms21228707 33218067 PMC7698958

[5] Wang Z Tu Z Xie X Cui H Kong KW Zhang L. Perilla frutescens leaf extract and fractions: Polyphenol composition, antioxidant, enzymes (α-glucosidase, acetylcholinesterase, and tyrosinase) inhibitory, anticancer, and antidiabetic activities. Foods. (2021) 10:315. 10.3390/foods10020315 33546380 PMC7913586

[6] Wang J Wang X Lv B Yuan W Feng Z Mi W. Effects of fructus akebiae on learning and memory impairment in a scopolamine-induced animal model of dementia. Exp Ther Med. (2014) 8:671–5. 10.3892/etm.2014.1775 25009638 PMC4079397

[7] Dong X Bai Y Xu Z Shi Y Sun Y Janaswamy S. Phlorotannins from *undaria pinnatifida* sporophyll: extraction, antioxidant, and anti-inflammatory activities. Mar Drugs. (2019) 17:434. 10.3390/md17080434 31344874 PMC6723497

[8] Hu J Zhang J Shan H Chen Z. Expression of floral mads-box genes in *sinofranchetia chinensis* (lardizabalaceae): implications for the nature of the nectar leaves. Ann Bot. (2012) 110:57–69. 10.1093/aob/mcs104 22652421 PMC3380600

[9] Zou S Yao X Zhong C Gao P Wang Z Huang H. Phenotypic characterization of *stauntonia obovatifoliola* hayata subsp. Urophylla germplasm: a potential new fruit crop. Genet ResourE Crop Ev. (2020) 67:1037–50. 10.1007/s10722-020-00885-9

[10] Peng X-b Gao W-l Hu D-q Ma F-f Fu L-g Deng Q. Chemical constituents from the aerial part of *stauntonia obovatifoliola* hayata subsp. Urophylla. Zhong Yao Cai. (2013) 36:1795–8. 10.13863/j.issn1001-4454.2013.11.041 24956822

[11] Kim Y Shin J Kim D-W Lee H-S Choi C. Complete chloroplast genome sequence of *stauntonia hexaphylla* (ranunculales: Lardizabalaceae), a species endemic to korea. Mitochondrial DNA B. (2021) 6:860–1. 10.1080/23802359.2021.1885320 33796658 PMC7971286

[12] Lu X Qiu F Pan X Li J Wang M Gong M. Simultaneous quantitative analysis of nine triterpenoid saponins for the quality control of stauntonia obovatifoliola hayata subsp. intermedia stems. J Sep Sci. (2014) 37:3632–40. 10.1002/jssc.201400771 25315436

[13] Thomas SS Cha Y-S Kim K-A. Perilla oil alleviates high-fat diet-induced inflammation in the colon of mice by suppressing nuclear factor-kappa b activation. J Med Food. (2020) 23:818–26. 10.1089/jmf.2019.4675 32552354

[14] Inoue Y Kitani Y Osakabe S Yamamoto Y Murata I Kanamoto I. The effects of gold kiwifruit intake timing with or without pericarp on postprandial blood glucose level. Nutrients. (2021) 13:2103. 10.3390/nu13062103 34205359 PMC8235107

[15] Zhang X Xu J Xu Z Sun X Zhu J Zhang Y. Analysis of antioxidant activity and flavonoids metabolites in peel and flesh of red-fleshed apple varieties. Molecules. (2020) 25:1968. 10.3390/molecules25081968 32340213 PMC7221745

[16] Alam MS Liang XF Liu L He S Kuang Y Hoseinifar SH. Growth and metabolic response of chinese perch to different dietary protein-to-energy ratios in artificial diets. Int J Mol Sci. (2019) 20:5983. 10.3390/ijms20235983 31795078 PMC6928951

[17] Schaub S Finger R Leiber F Probst S Kreuzer M Weigelt A. Plant diversity effects on forage quality, yield and revenues of semi-natural grasslands. Nat Commun. (2020) 11:768. 10.1038/s41467-020-14541-4 32034149 PMC7005841

[18] Kazimierska K Biel W Witkowicz R. Mineral composition of cereal and cereal-free dry dog foods versus nutritional guidelines. Molecules. (2020) 25:5173. 10.3390/molecules25215173 33172044 PMC7664208

[19] Lebaka VR Wee YJ Ye W Korivi M. Nutritional composition and bioactive compounds in three different parts of mango fruit. Int J Environ Res Public Health. (2021) 18:1073. 10.3390/ijerph18020741 33467139 PMC7830918

[20] Njoku NE Ubbaonu CN Alagbaoso SO Eluchie CN Umelo MC. Amino acid profile and oxidizable vitamin content of *synsepalum dulcificum* berry (miracle fruit) pulp. Food Sci Nutr. (2015) 3:252–6. 10.1002/fsn3.213 25988000 PMC4431793

[21] Rana ZH Alam MK Akhtaruzzaman M. Nutritional composition, total phenolic content, antioxidant and α-amylase inhibitory activities of different fractions of selected wild edible plants. Antioxid. (2019) 8:203. 10.3390/antiox8070203 31266183 PMC6680810

[22] He J Dong Y Liu X Wan Y Gu T Zhou X. Comparison of chemical compositions, antioxidant, and anti-photoaging activities of paeonia suffruticosa flowers at different flowering stages. Antioxid. (2019) 8:345. 10.3390/antiox8090345 31480512 PMC6770142

[23] Gong ES Li B Li B Podio NS Chen H Li T. Identification of key phenolic compounds responsible for antioxidant activities of free and bound fractions of blackberry varieties' extracts by boosted regression trees. J Sci Food Agric. (2022) 102:984–94. 10.1002/jsfa.11432 34302364

[24] Botas J Fernandes Â Barros L Alves MJ Carvalho AM Ferreira ICFR. A comparative study of black and white allium sativum l: nutritional composition and bioactive properties. Molecules. (2019) 24:2194. 10.3390/molecules24112194 31212722 PMC6600231

[25] Zhu H Liu S Yao L Wang L Li C. Free and bound phenolics of buckwheat varieties: Hplc characterization, antioxidant activity, and inhibitory potency towards α-glucosidase with molecular docking analysis. Antioxid. (2019) 8:606. 10.3390/antiox8120606 31795516 PMC6943536

[26] Aparnak P Saberivand A. Effects of curcumin on canine semen parameters and expression of nox5 gene in cryopreserved spermatozoa. Vet Res Forum. (2019) 10:221–6. 31737231 10.30466/vrf.2019.76137.2015 PMC6828172

[27] Desai AS Brennan MA Guo X Zeng X-A Brennan CS. Fish protein and lipid interactions on the digestibility and bioavailability of starch and protein from durum wheat pasta. Molecules. (2019) 24:839. 10.3390/molecules24050839 30818770 PMC6429422

[28] Liao H Banbury L. Different proportions of huangqi (*radix astragali mongolici*) and honghua (*flos carthami*) injection on α-glucosidase and α-amylase activities. Evid Based Complementary Altern Med. (2015) 2015:785193. 10.1155/2015/785193 25873983 PMC4385629

[29] Ellman GL Courtney KD Andres VJr Featherstone RM. A new and rapid colorimetric determination of acetylcholinesterase activity. Biochem Pharmacol. (1961) 7:88–95. 10.1016/0006-2952(61)90145-9 13726518

[30] Sarker U Oba S. Antioxidant constituents of three selected red and green color *amaranthus* leafy vegetable. Sci Rep. (2019) 9:18233. 10.1038/s41598-019-52033-8 31796754 PMC6890792

[31] Wojdyło A Nowicka P Turkiewicz IP Tkacz K Hernandez F. Comparison of bioactive compounds and health promoting properties of fruits and leaves of apple, pear and quince. Sci Rep. (2021) 11:20253. 10.1038/s41598-021-99293-x 34642358 PMC8511160

[32] Kühne F Biedermann M Eicher A Felder F Sander S Schmidt R. Characterisation of elastomers as food contact materials-part 1: quantification of extractable compounds, swelling of elastomers in food simulants and release of elements. Molecules. (2021) 26:509. 10.3390/molecules26020509 33478042 PMC7835956

[33] Wang Yujuan XW He X Duan W Huang W Gong C. Differential comparison among different geographical provenances of *stauntonia urophylla*. J Cent South Univ. (2015) 35:55–60.

[34] Reig G Iglesias I Gatius F Alegre S. Antioxidant capacity, quality, and anthocyanin and nutrient contents o f several peach cultivars [prunus persica (l) batsch] grown in spain. J Agric Food Chem. (2013) 61:6344–57. 10.1021/jf401183d 23713711

[35] Chang L Xu D Zhu J Ge G Kong X Zhou Y. Herbal therapy for the treatment of acetaminophen-associated liver injury: recent advances and future perspectives. Front Pharmacol. (2020) 11:313. 10.3389/fphar.2020.00313 32218738 PMC7078345

[36] Zheng XQ Wang JT Liu XL Sun Y Zheng YJ Wang XJ. Effect of hydrolysis time on the physicochemical and functional properties of corn glutelin by protamex hydrolysis. Food Chem. (2015) 172:407–15. 10.1016/j.foodchem.2014.09.080 25442571

[37] Afzal S Chaudhry BA Ahmad A Uzair M Afzal K. Antioxidant, acetylcholinesterase, butyrylcholinesterase, and α-glucosidase inhibitory activities of *corchorus depressus*. Pharmacogn Mag. (2017) 13:647–51. 10.4103/pm.pm_95_17 29200727 PMC5701405

[38] Ruiz-Ruiz JC Moguel-Ordoñez YB Matus-Basto AJ Segura-Campos MR. Antidiabetic and antioxidant activity of stevia rebaudiana extracts (var. Morita) and their incorporation into a potential functional bread. J Food Sci Technol. (2015) 52:7894–903. 10.1007/s13197-015-1883-3 26604361 PMC4648875

[39] Bahar-Fuchs A Martyr A Goh AM Sabates J Clare L. Cognitive training for people with mild to moderate dementia. Cochrane Database Syst Rev. (2019) 3:Cd013069. 10.1002/14651858.CD013069.pub2 30909318 PMC6433473

[40] Gonçalves S Medronho J Moreira E Grosso C Andrade PB Valentão P. Bioactive properties of chamaerops humilis l: antioxidant and enzyme inhibiting activities of extracts from leaves, seeds, pulp and peel. 3 Biotech. (2018) 8:88. 10.1007/s13205-018-1110-9 29430350 PMC5799110

[41] Sinan KI Chiavaroli A Orlando G Bene K Zengin G Cziáky Z. Evaluation of pharmacological and phytochemical profiles piptadeniastrum africanum (hookF) brenan stem bark extracts. Biomolecules. (2020) 10:516. 10.3390/biom10040516 32231150 PMC7226170

[42] Suleria HAR Barrow CJ Dunshea FR. Screening and characterization of phenolic compounds and their antioxidant capacity in different fruit peels. Foods. (2020) 9:1206. 10.3390/foods9091206 32882848 PMC7556026

[43] Drapal M Barros de Carvalho E Ovalle Rivera TM Becerra Lopez-Lavalle LA Fraser PD. Capturing biochemical diversity in cassava (manihot esculenta crantz) through the application of metabolite profiling. J Agric Food Chem. (2019) 67:986–93. 10.1021/acs.jafc.8b04769 30557498 PMC6346375

[44] Niu L Hou Y Jiang M Bai G. The rich pharmacological activities of *magnolia officinalis* and second ary effects based on significant intestinal contributions. J Ethnopharmacol. (2021) 281:114524. 10.1016/j.jep.2021.114524 34400262

[45] Rusu ME Fizeşan I Pop A Gheldiu A-M Mocan A Crişan G. Enhanced recovery of antioxidant compounds from hazelnut (corylus avellana l) involucre based on extraction optimization: phytochemical profile and biological activities. Antioxid. (2019) 8:460. 10.3390/antiox8100460 31597384 PMC6826866

[46] Singh R Singh S Kumar S Arora S. Evaluation of antioxidant potential of ethyl acetate extract/fractions of acacia auriculiformis a. Cunn Food Chem Toxicol. (2007) 45:1216–23. 10.1016/j.fct.2007.01.002 17336438

[47] Nxumalo CI Ngidi LS Shandu JSE Maliehe TS. Isolation of endophytic bacteria from the leaves of *anredera cordifolia* cix1 for metabolites and their biological activities. BMC Complement Altern. (2020) 20:300. 10.1186/s12906-020-03095-z 33028279 PMC7541265

[48] Pervin M Hasnat MA Lee YM Kim DH Jo JE Lim BO. Antioxidant activity and acetylcholinesterase inhibition of grape skin anthocyanin (gsa). Molecules. (2014) 19:9403–18. 10.3390/molecules19079403 24995924 PMC6271686

[49] Wu P Li F Zhang J Yang B Ji Z Chen W. Phytochemical compositions of extract from peel of hawthorn fruit, and its antioxidant capacity, cell growth inhibition, and acetylcholinesterase inhibitory activity. BioMed Central. (2017) 17:151. 10.1186/s12906-017-1662-y 28284186 PMC5346202

